# The GRAS Salts of Na_2_SiO_3_ and EDTA-Na_2_ Control Citrus Postharvest Pathogens by Disrupting the Cell Membrane

**DOI:** 10.3390/foods12122368

**Published:** 2023-06-15

**Authors:** Juan Zhao, Yuqing Wang, Qianyi Liu, Shuqi Liu, Hui Pan, Yunjiang Cheng, Chaoan Long

**Affiliations:** 1 National Key Laboratory for Germplasm Innovation & Utilization of Horticultural Crops, National R&D Center for Citrus Preservation, National Centre of Citrus Breeding, Huazhong Agricultural University, Wuhan 430070, China; zhaojuan@mail.hzau.edu.cn (J.Z.);; 2 Shenzhen Institute of Nutrition and Health, Huazhong Agricultural University, Wuhan 430070, China; 3 Shenzhen Branch, Guangdong Laboratory for Lingnan Modern Agriculture, Genome Analysis Laboratory of the Ministry of Agriculture, Agricultural Genomics Institute at Shenzhen, Chinese Academy of Agricultural Sciences, Shenzhen 518120, China

**Keywords:** GRAS salts, EC_50_, postharvest pathogens, spore germination, lipid droplets

## Abstract

Sodium silicate (Na_2_SiO_3_) and ethylenediaminetetraacetic acid disodium salt (EDTA-Na_2_) are inorganic salts classified as ‘Generally Recognized as Safe’ (GRAS) compounds with great advantages in controlling various pathogens of postharvest fruits and vegetables. Here, we determined the median effective concentration (EC_50_) of Na_2_SiO_3_ (0.06%, 0.05%, 0.07% and 0.08%) and EDTA-Na_2_ (0.11%, 0.08%, 0.5%, and 0.07%) against common pathogens affecting postharvest citrus fruit, including *Penicillium digitatum*, *Penicillium italicum*, *Geotrichum citri-aurantii*, and *Colletotrichum gloeosporioides*. Na_2_SiO_3_ and EDTA-Na_2_ treatments at the EC_50_ decreased the spore germination rate, visibly disrupted the spore cell membrane integrity, and significantly increased the lipid droplets (LDs) of the four postharvest pathogens. Moreover, both treatments at EC_50_ significantly reduced the disease incidence of *P. italicum* (by 60% and 93.335, respectively) and *G. citri-aurantii* (by 50% and 76.67%, respectively) relative to the control. Furthermore, Na_2_SiO_3_ and EDTA-Na_2_ treatment resulted in dramatically lower disease severity of the four pathogens, while also demonstrating no significant change in citrus fruit quality compared with the control. Therefore, Na_2_SiO_3_ and EDTA-Na_2_ present a promising approach to control the postharvest diseases of citrus fruit.

## 1. Introduction

Citrus fruits such as orange, lemon, lime, mandarin orange, and pomelo, which are cultivated in more than 130 countries, are in high demand worldwide [1]. Citrus fruits are a rich source of vitamin C, dietary fiber, phenolic acids, and other bioactive compounds [2,3]. According to FAO, global citrus production exceeded 147 million tons in 2022, playing an essential role in the development of the global agricultural economy (http://www.fao.org/faostat/en/?#data/QC, accessed on 12 December 2020). However, various citrus diseases cause huge economic losses and seriously affect the development of the citrus industry [4]. *Penicillium digitatum*, *Penicillium italicum*, and *Geotrichum citri-aurantii* are three pathogens responsible for causing the greatest post-harvest loss of citrus fruit. They can infect the fruit through wound during transport, packing, and post-harvest storage [5]. Anthracnose is another serious citrus disease caused by *Colletotrichum* spp., and can infect citrus fruit not only in the field but also during post-harvest storage, which has serious negative impacts on the yield and quality of citrus fruit [6,7]. Overall, these post-harvest losses were estimated to reach 20–50% in developing countries and 10–20% in developed countries in the fresh and juice citrus industries [8].

Chemical fungicides, including thiabendazole, imazalil, fludioxonil, pyrimethanil, and sodium o-phenylphenate, have been used for continuous control of citrus post-harvest diseases for many years [9]. However, no chemical fungicide has shown satisfactory performance in controlling *G. citri-aurantii*. It has been reported that the ergosterol demethylation inhibitor (DMI) triazole propiconazole (PCZ) is the only highly effective fungicide for post-harvest sour rot after the prohibition of guazatine in the European Union (EU) [10]. Furthermore, due to its potential harmful effects on human health, this active substance was prohibited by the EU on 28 November 2018. Consequently, there is a lack of effective and safe fungicides to control sour rot in Europe [11]. Currently, there are increasing concerns about the side effects of fungicide residues, environmental pollution, and pathogen fungicide resistance [1].

To solve these problems, some natural alternatives have been proposed to control citrus post-harvest diseases, such as certain microorganisms as biocontrol agents [12,13,14,15], essential oils [16,17,18], and plant extracts [19,20]. However, the commercial use of these alternatives has been seriously hindered by the low efficacy or instability of the formulated products [21]. Therefore, there is an urgent need for safe and commercially viable strategies for the control of citrus post-harvest diseases.

Organic and inorganic salts classified as ‘Generally Recognized as Safe’ (GRAS) compounds exhibit great advantages in post-harvest disease control, such as high solubility in water, easy synthesis, and wide availability at relative low costs. More importantly, the United States Food and Drug Administration (US FDA) has exempted the residue detection of these compounds on all agricultural commodities [22,23]. Furthermore, they are also approved to be widely used in food without safety concerns by international regulators. Therefore, GRAS salts may be a promising and safe alternative to fungicides for controlling post-harvest diseases in fresh citrus fruit [24].

In previous studies, several GRAS salts have been demonstrated to effectively control citrus post-harvest diseases. For example, alkaline (alEW) and acidic (acEW) electrolyzed water of sodium metabisulfite (SM), potassium sorbate (PS), potassium carbonate (PC), and sodium chloride (SC) have direct impacts on the radial growth, conidial germination, germ tube elongation, and morphological changes of green and blue molds, without negative effects on the quality properties of ‘Valencia’ late orange [25]. Potassium silicate (PSi), sodium benzoate (SB), sodium methylparaben (SMP), and sodium ethylparaben (SEP) can effectively reduce the incidence and severity of both green and blue molds [26,27,28]. In addition, the curative activity of PS, SMP (200 mM), SEP (200 mM), or SB (3% *w*/*v*) was found to reduce the sour rot incidence and severity by up to 90% [11,29]. Moreover, coatings containing 2% PS, 2% SB, and 2% PSi are the most effective to reduce anthracnose severity by up to 70% on mandarins [7]. With great progress in the relevant research fields, more and more GRAS salts have been commercially applied to citrus industries in Western countries [5]. However, there are fewer studies on the application of GRAS salts in China, where citrus post-harvest diseases are still mostly controlled by traditional chemical fungicides. Moreover, only a small proportion of GRAS salts have been used and there is a serious lack of broad-spectrum antifungal GRAS salts.

By referring to the relevant literature, this study used the Oxford cup method to evaluate the inhibitory activity of GRAS salts on different pathogens by determining the diameter of the inhibition zone (IZ), and evaluated the activity of the screened GRAS salts against *Penicillium digitatum*, *Penicillium italicum*, *Geotrichum citri-aurantii,* and *Colletotrichum gloeosporioides in vitro*, as well as explored the possible inhibition mechanisms of the selected GRAS salts. Furthermore, the ability of the selected GRAS salts to control the four citrus post-harvest diseases was assessed by in vivo experiments with artificial pathogen inoculation on mandarins and oranges. The findings will provide an important reference for developing asepsis and high-efficiency antiseptic and freshening agents for citrus fruit.

## 2. Materials and Methods

### 2.1. Fungal Strain, Citrus Fruit and GRAS Salts

*Penicillium digitatum* (referred to as P44) and *Penicillium italicum* (referred to as B3) were obtained from the University of Bari Aldo Moro (donated by Professor Antonio Ippolito). *Geotrichum citri-aurantii* (referred to as AY-1) and *Colletotrichum gloeosporioides* (referred to as NF28) were isolated from Newhall navel orange in Anyuan city, Jiangxi Province of China in 2012. They were activated on PDA medium (2% glucose and 1.5% agar in an infusion from potatoes) at 25 °C for 3–5 days, and identified and purified three times as described in our previous study [30]. All pathogens were identified with ITS primers again.

Valencia orange fruits were harvested from the orchard (Huazhong Agricultural University). The GRAS salts were of food grade and purchased from Zhejiang Yinuo Biological Technology Co., Ltd. (Lanxi, China)

**Figure 1:**
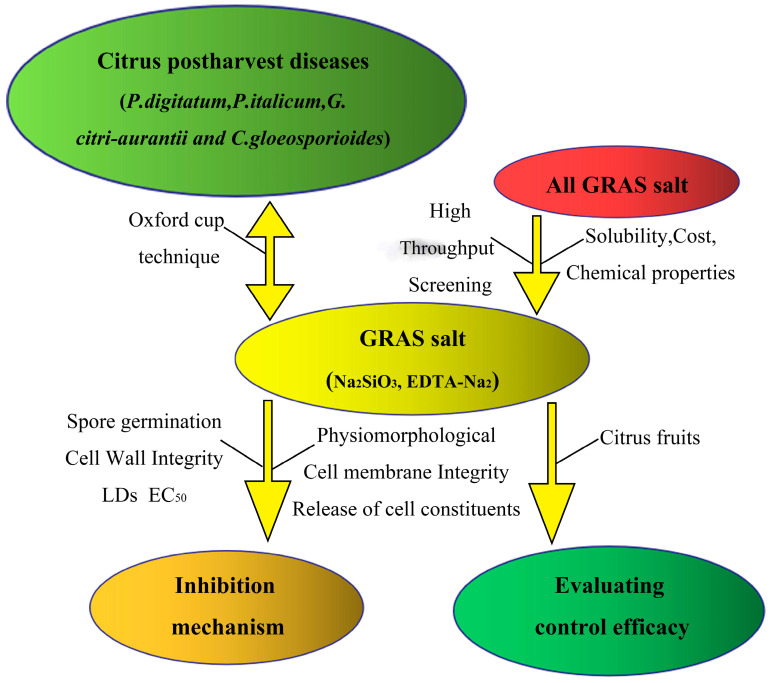
Technical route of this study. SS: Na_2_SiO_3_, EA: EDTA-Na_2_, LDs: lipid droplets; EC_50_: the median effective concentration.

The experimental scheme was designed as shown in Figure 1.

### 2.2. High-Throughput Screening of GRAS Salts for Citrus Postharvest Diseases

There are hundreds of GRAS salts that can be used for controlling citrus post-harvest diseases. Here, we first collected the data of all GRAS salts according to the United States Food and Drug Administration (US FDA), and then screened the most appropriate GRAS salts of high solubility in water, steady chemical property, no unpleasant smell, no stimulation of the skin, no harm, no toxicity, and wide availability at relatively low costs.

### 2.3. Evaluation of Antifungal Activity In Vitro

The antifungal activity of GRAS salts against *P. digitatum*, *P. italicum*, *G. citri-aurantii*, and *C. gloeosporioides* was evaluated using the Oxford cup technique with some modifications [31]. Briefly, 100 μL spore suspension (1.0 × 10^6^ CFU mL^−1^) of these pathogens were added into PDA medium at 40 °C to avoid scalding to death. Then, sterilized Oxford cups were inserted gently in the center of the PDA medium, and 250 μL GRAS salt solution (1%) was dropped. The diameter of the inhibition zone (IZ) was measured after 5 d of incubation at 25 °C.

After selection of GRAS salts with obvious IZ, the inhibition rate of every GRAS salt at 1% was determined and calculated. Then, the EC_50_ of GRAS salts with a broad spectrum of antifungal function to the four citrus post-harvest pathogens was detected, and the inhibitory effect of these salts on the growth of hyphae was determined with the agar dilution method as described in a previous study [32].

The PDA medium contained different concentrations (0, 0.02%, 0.04%, 0.06%, 0.08%, and 0.16%) of sodium silicate (Na_2_SiO_3_, SS), and ethylenediaminetetraacetic acid disodium salt (EDTA-Na_2_, EA) was prepared into final concentrations of 0, 0.03%, 0.06%, 0.12%, 0.24%, and 0.48%. Then, 2.5 μL of fresh spore suspension (10^6^ CFU mL^−1^) was deposited in the center of the culture dish and incubated at 25 °C in a constant-temperature incubator (Bo er Si, BES600SH). Finally, each measurement consisted of three 90-mm Petri dishes. The logarithm of the concentration of 10 was used as the abscissa and the probability value corresponding to the inhibition rate was used as the ordinate to obtain the regression equation and the correlation coefficient R^2^ for each test concentration. The EC_50_ value and its 95% confidence interval were calculated based on the probability value corresponding to 50% inhibition [33].

### 2.4. Effect of Na_2_SiO_3_ and EDTA-Na_2_ on Spore and Hyphal Morphology

First, 50 μL of 10^6^ CFU mL^−1^ fresh spore suspension (*P. Digitatum*, *P. italicum*, *G. citri-aurantii*, and *C. gloeosporioides*) was prepared and added into 50 mL PDB. After shaking for 12 h at 25 °C and 150 r min^−1^, Na_2_SiO_3_ and EDTA-Na_2_ were added into the PDB medium to the final EC_50_ concentration, respectively. The PDB without the addition of any pathogen was used as the negative control. All 12 samples continued to be incubated for 12 h. Finally, the morphology of spores and hyphae was observed via light microscopy (Nikon Eclipse E100, Nikon corporation, Tokyo, Japan).

### 2.5. Effect of Na_2_SiO_3_ and EDTA-Na_2_ on Spore Germination

The spore suspension of every sample was prepared using the same method as in the previous section. The germination of spores was recorded via light microscopy. The rate of spore germination (RSG) = (the number of germination spore/the number of total spores) × 100%.

### 2.6. Cell Wall Integrity, Cell Membrane Integrity, and Lipid Droplet Accumulation Assays

The spore suspension and the PDB medium with Na_2_SiO_3_ and EDTA-Na_2_ were prepared as described in Section 2.4 above. Then, 100 μL spore suspensions (1 × 10^6^ CFU mL^−1^) of four citrus post-harvest pathogens were added in the prepared PDB medium, and these suspensions were shaken for 8 h at 25 °C, centrifuged at 8000 r min^−1^ for 10 min [34], and the supernatant was removed.

Cell wall integrity assay: Calcofluor white (CFW, Coolaber, Beijing, China) was used to show the cell wall because of the special etch reactive. The cell wall integrity of spores and hyphae was determined with a modified protocol [34]. Spores were dyed with 10 μL CFW containing 10% KOH [32], and then observed using a fluorescence microscope (Nikon Eclipse 90i).

Cell membrane integrity assay: The cell membrane integrity of spores and hyphae was determined with a modified method [32]. Spores and hyphae were collected and dyed with 10 μg mL^−1^ propidium iodide (PI, (Coolaber Technology Co., Ltd., Beijing, China)). They were stained at 37 °C for 30 min [35] and the floating dye was washed off with PBS for three times. All samples were observed using a fluorescence microscope (Nikon Eclipse 90i).

Lipid droplet (LD) accumulation assay: LDs of all samples were observed according to the modified protocol developed in a previous study [32]. Fresh spores and hyphae were dyed with the Nile Red solution (Coolaber, Beijing, China) for 3–6 min, then washed twice with 0.1 × PBS buffer. LDs were observed using a fluorescence microscope.

### 2.7. Release of Cell Components

Detection of the cell components for these citrus post-harvest pathogens after EC_50_ Na_2_SiO_3_ and EDTA-Na_2_ treatment was performed with a modified method [34]. The spore suspensions were prepared and treated as described in Section 2.4 above; however, all 12 samples only continued to be incubated for 2 h after adding Na_2_SiO_3_ and EDTA-Na_2_ at the EC_50_. Finally, the fungal suspensions were centrifuged at 8000 r min^−1^ for 10 min, and then detected at 260 nm using a UV-spectrophotometer (UV-1500, AOE INSTRUMENTS (Shanghai, China) Co., Ltd.).

### 2.8. Fruit Decay Test

Healthy unwounded citrus fruit were soaked in 2% (*v*/*v*) sodium hypochlorite for 2 min and air-dried after washing twice with distilled water. Then, three wounds (4 mm deep and 4 mm wide) were created at equal spaces in the equator with an inoculating needle. The optimal concentrations for in vivo experiments were determined according to the *in vitro* results. Each wound of the fruit was inoculated with 20 μL spore suspension (10^7^ CFU mL^−1^) of the four citrus post-harvest pathogens, respectively [35]. After air-drying of the wounds, the fruits were dipped in 0 (control) and EC_95_ Na_2_SiO_3_ and EDTA-Na_2_ solution for 2 min and then stored in a plastic crisper with wet tissue at room temperature. The disease incidence and lesion size were determined after 7 d. The disease incidence (DI) = (decaying wounds number/total wounds number) × 100%. Every experimental group included five fruits, and was repeated three times.

### 2.9. Fruit Quality Evaluation In Vivo

To verify the practical value of Na_2_SiO_3_ and EDTA-Na_2_ in the citrus industry, long-term storage experiments were performed in a storehouse in Zhijiang city, Hubei province in China. The fruits were soaked in EC_95_ Na_2_SiO_3_ and EDTA-Na_2_ solution for 2 min, with tap-water as the control. Treated fruits were stored for three months under natural conditions, during which the fruit quality (fruit weight-loss rate, soluble solid, titration acid contents, and VC) was constantly monitored. Each treatment contained 300 fruit in three replicates.

### 2.10. Statistical Analysis

All experiments were conducted with a completely randomized design and repeated three times. SPSS 26.0 statistical software was used to analyze the data. The results were reported as the average value of the three replicates. Moreover, the standard error and significant differences were calculated with one-way ANOVA followed by Duncan’s Multiple Range test (*p* < 0.05).

## 3. Results

### 3.1. Inhibition Rates of 17 GRAS Salts (1%)

After screening of 42 GRAS salts with the potential to control citrus post-harvest diseases according to the above-mentioned criteria, 17 GRAS salts were found to exhibit different degrees of inhibitory effect on the growth of *P. Digitatum*, *P. italicum*, *G. citri-aurantii* and *C. gloeosporioides* (Table 1). The inhibition rate of Sodium silicate (Na_2_SiO_3_, 1%) and Ethylenediaminetetraacetic acid disodium salt (EDTA-Na_2_, 1%) was 100% on *P. italicum, G. citri-aurantii*, and *C. gloeosporioides*, and 100% and 87.90% on *P. digitatum*, respectively.

### 3.2. Digital Photography of Na_2_SiO_3_ and EDTA-Na_2_ against Four Postharvest Pathogens

**Figure 2:**
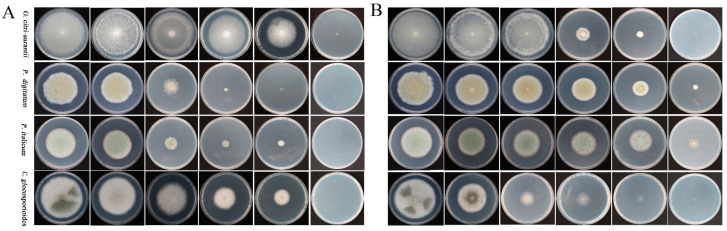
Effects of different concentrations of Na_2_SiO_3_ ((**A**); 0, 0.02%, 0.04%, 0.06%, 0.08%, and 0.16%) and EDTA-Na_2_ ((**B**); 0, 0.03%, 0.06%, 0.12%, 0.24%, and 0.48%) on the hyphal growth of *P. digitatum*, *P. italicum*, *G. citri-aurantii*, and *C. gloeosporioides*.

As shown in Figure 2, Na_2_SiO_3_ and EDTA-Na_2_ inhibited the hyphal growth of four pathogens in a concentration-dependent manner (Figure 2). Table 2 shows that the EC_50_ of Na_2_SiO_3_ was 0.06%, 0.05%, 0.07%, and 0.08%, and that of EDTA-Na_2_ was 0.11%, 0.08%, 0.5%, and 0.07% for *G. citri-aurantii*, *P. digitatum*, *P. italicum*, and *C. gloeosporioides*, respectively.

### 3.3. Effect of Na_2_SiO_3_ and EDTA-Na_2_ on the Spore and Hyphal Morphology

**Figure 3:**
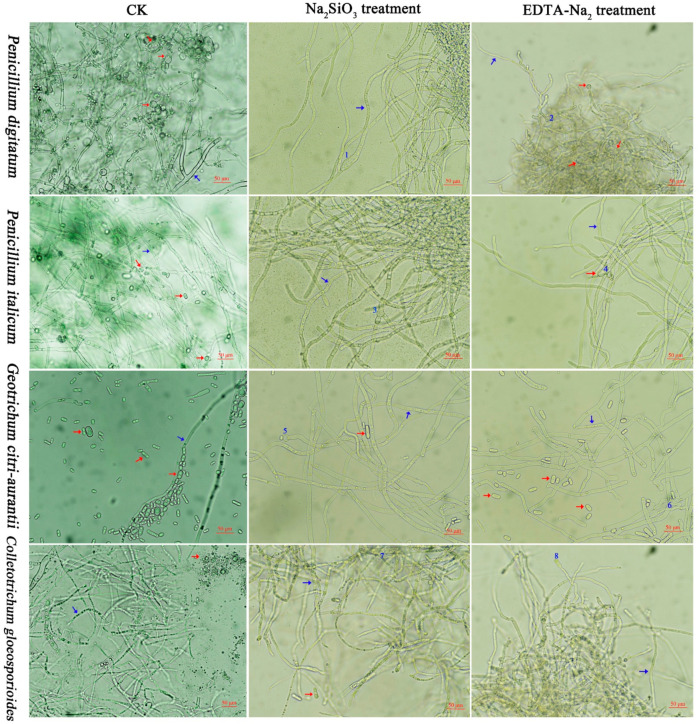
Number of *P. digitatum*, *P. italicum*, *G. citri-aurantii*, and *C. gloeosporioides* spores under Na_2_SiO_3_ and EDTA-Na_2_ treatment. The scale bar in all photos is 50 µm. (Spores (red arrow), mycelium (blue arrow)).

As shown in Figure 3, the number of spores decreased after Na_2_SiO_3_ and EDTA-Na_2_ treatment (red arrow); however, non-treated hyphae of the four pathogens showed a normal morphology with clear boundaries and loose arrangement. The hyphae were tightly aggregated and adhered to each other under Na_2_SiO_3_ and EDTA-Na_2_ treatment. Moreover, Na_2_SiO_3_ and EDTA-Na_2_ treatment caused abnormal bulges and swelling on the fungal hyphae (blue arrow and blue numbers of 1, 2, 3, 4, 5, 6, 7 and 8). In general, Na_2_SiO_3_ and EDTA-Na_2_ treatment obviously disrupted the fungal spores and hyphae of *P. digitatum*, *P. italicum*, *G. citri-aurantii*, and *C. gloeosporioides*.

### 3.4. Effect of Na_2_SiO_3_ and EDTA-Na_2_ on Spore Germination

**Figure 4:**
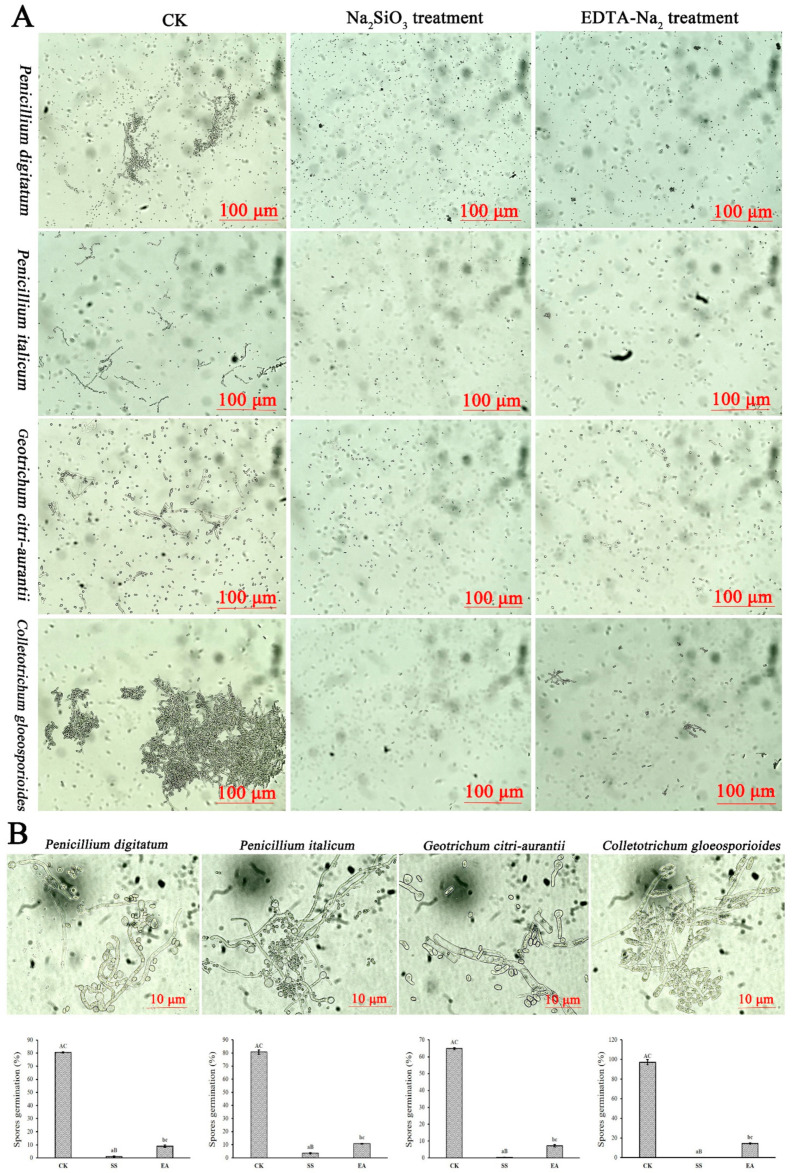
Effects of EC_50_ Na_2_SiO_3_ and EDTA-Na_2_ treatments on the spore germination of *P. digitatum*, *P. italicum*, *G. citri-aurantii*, and *C. gloeosporioides*. (**A**) Inhibition efficacy of EC_50_ Na_2_SiO_3_ and EDTA-Na_2_ on the spore germination of *P. digitatum*, *P. italicum*, *G. citri-aurantii*, and *C. gloeosporioides*; (**B**) Percentage of spore germination. The scale bar of B is 10 µm, and that of other photos is 100 µm. Error bar means the standard error. Different lower-case letters are significantly different (*p* < 0.05), different upper-case letters are significantly different (*p* < 0.01).

As shown in Figure 4, Na_2_SiO_3_ and EDTA-Na_2_ remarkably inhibited the spore germination of four pathogens (Figure 4A). The germination rate of *P. digitatum*, *P. italicum*, *G. citri-aurantii*, and *C. gloeosporioides* was 3.46%, 0.94%, 0.23%, and 0 under the EC_50_ Na_2_SiO_3_ treatment, and 10.82%, 8.97%, 7.16%, and 14.47% under EC_50_ EDTA-Na_2_ treatment, respectively which were significantly decreased compared with that of the control (80.71%, 80.48%, 64.87%, and 97.11%) (*p* < 0.05) (Figure 4B). Obviously, Na_2_SiO_3_ treatment resulted in a lower germination rate than the EDTA-Na_2_ treatment. Moreover, Na_2_SiO_3_ and EDTA-Na_2_ also showed certain inhibitory effects on the germ tube elongation (Figure 4A).

### 3.5. Effect of Na_2_SiO_3_ and EDTA-Na_2_ on Cell Wall Integrity and Lipid Droplet Accumulation

**Figure 5:**
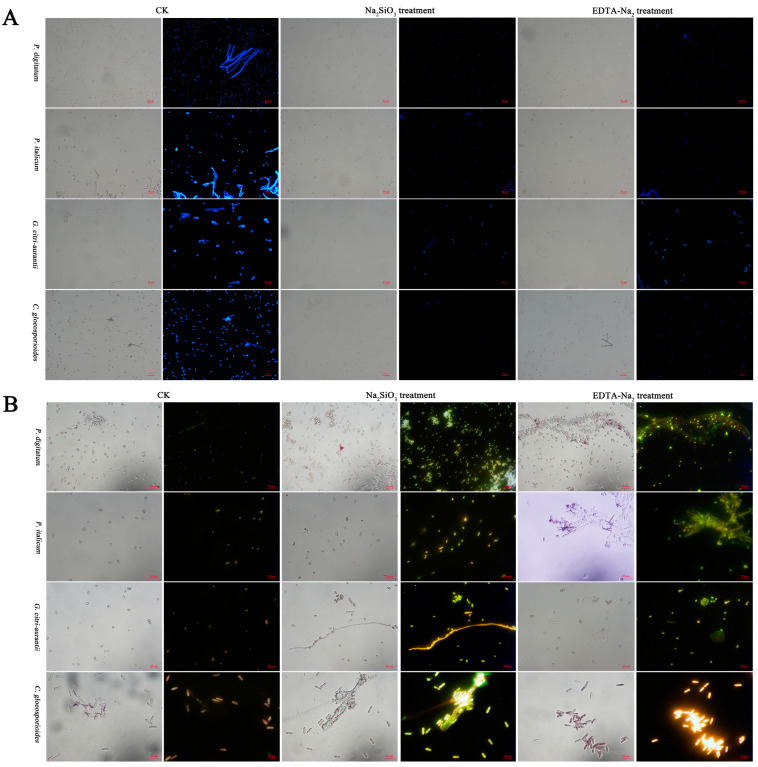
Fluorescence microscopy images of EC_50_ Na_2_SiO_3_ and EDTA-Na_2_ on spores of *P. digitatum*, *P. italicum*, *G. citri-aurantii*, and *C. gloeosporioides*. (**A**) Spores under bright field and propidium iodide (CFW); (**B**) LDs under bright field and propidium iodide (Nile Red). The scale bar in the photos is 20 µm.

As shown in Figure 5A, the blue fluorescence brightness of spore cell walls of the four pathogens generally remained unchanged after Na_2_SiO_3_ and EDTA-Na_2_ treatment compared with that of the control in CFW-staining assay, suggesting that Na_2_SiO_3_ and EDTA-Na_2_ treatments caused no obvious damage to the cell wall integrity.

The accumulation of LD in spores and hyphae was observed using LD staining with Nile red solution (Figure 5B). The fluorescence intensity of the experiment group was obviously higher than that of the control, because of a significant increase in LD biogenesis under Na_2_SiO_3_ and EDTA-Na_2_ treatments.

### 3.6. Effect of Na_2_SiO_3_ and EDTA-Na_2_ on Cell Membrane Integrity

**Figure 6:**
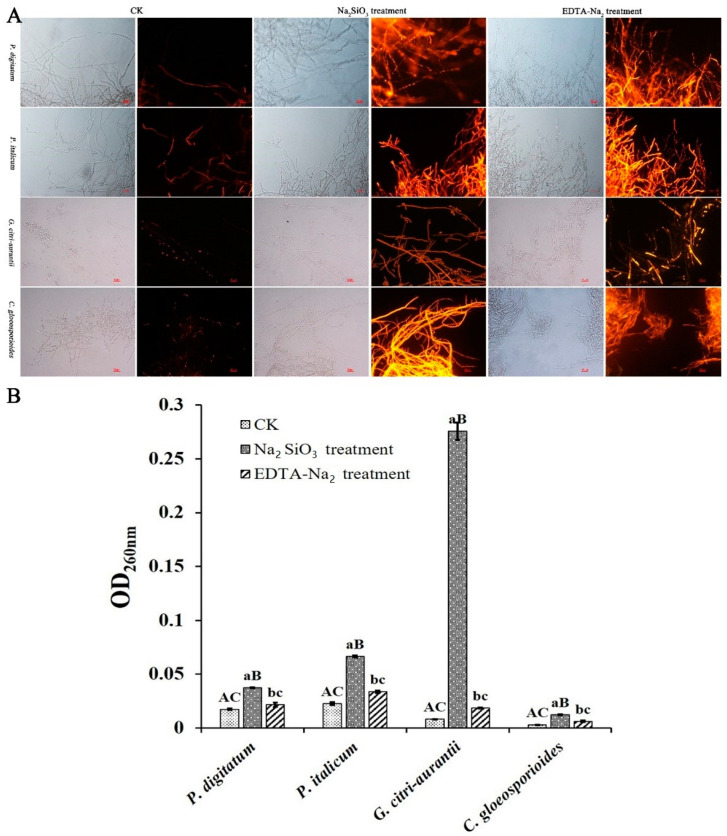
Effect of EC_50_ Na_2_SiO_3_ and EDTA-Na_2_ on hyphal cell membrane integrity of *P. digitatum*, *P. italicum*, *G. citri-aurantii*, and *C. gloeosporioides*. (**A**) Fluorescence microscopy image of hyphae under bright field and propidium iodide (PI), the scale bar in the photos is 25 µm; (**B**) Effect of cinnamic acid on nucleic acid leakage. The results were reported by the average value. Error bar means the standard error. Different lower-case letters are significantly different (*p* < 0.05), different upper-case letters are significantly different (*p* < 0.01).

As shown in Figure 6A, Na_2_SiO_3_ and EDTA-Na_2_ treatments led to clear red fluorescence compared with the control, indicating that the two treatments disrupt the hyphal cell membrane of pathogens.

### 3.7. Effect of Na_2_SiO_3_ and EDTA-Na_2_ on the Nucleic Acid Leakage

The nucleic acid concentration was determined to further confirm the disruption of cell membrane integrity. The nucleic acid concentration increased under Na_2_SiO_3_ and EDTA-Na_2_ treatments. As shown in Figure 6B, the OD_260_ values of *P. digitatum, P. italicum, G. citri-aurantii,* and *C. gloeosporioides* were 0.037, 0.067, 0.28, and 0.01 after Na_2_SiO_3_ treatment, and 0.022, 0.034, 0.019, and 0.006 after EDTA-Na_2_ treatment, respectively, which were all apparently higher than those of the control (0.017, 0.023, 0.008, and 0.003, respectively). These results indicated that Na_2_SiO_3_ and EDTA-Na_2_ disrupted the cell membrane, resulting in a massive leakage of nucleic acid.

### 3.8. Pathogen Inhibition Ability of Na_2_SiO_3_ and EDTA-Na_2_ In Vivo

**Figure 7:**
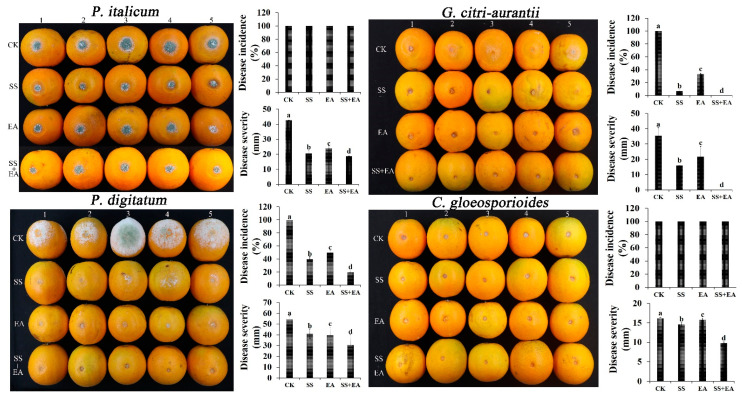
Disease incidence and disease severity of *P. digitatum*, *P. italicum*, *G. citri-aurantii* and *C. gloeosporioides* under EC_50_ Na_2_SiO_3_ and EDTA-Na_2_ treatments. SS: Na_2_SiO_3_, EA: EDTA-Na_2_, SS + SA: Na_2_SiO_3_ + EDTA-Na_2_. Each group contained five fruit. The results are reported by the average value. Error bar means the standard error. Different lower-case letters are significantly different (*p* < 0.05).

The inhibition ability of Na_2_SiO_3_ and EDTA-Na_2_ on citrus fruit post-harvest pathogens is shown in Figure 7. After treatment with Na_2_SiO_3_ and EDTA-Na_2_, the decay rates caused by *P. italicum* (40 ± 3.64% and 50 ± 2.83%) and *G. citri-aurantii* (6.67 ± 2.46% and 33.33 ± 1.63%) were lower than that in the control (100% and 100%). Furthermore, the disease severity of *P. digitatum* (20.67 ± 3.64 mm and 24.02 ± 2.83 mm), *P. italicum* (41.08 ± 4.31 mm and 39.76 ± 7.23 mm), *G. citri-aurantii* (16.00 ± 0.02 mm and 21.82 ± 6.67 mm), and *C. gloeosporioides* (14.64 ± 0.45 mm and 15.79 ± 0.47 mm) was also dramatically reduced compared with that in the control (42.68 ± 0.64 mm, 54.50 ± 3.10 mm, 35.34 ± 5.45 mm, and 16.19 ± 0.27 mm). Moreover, a mixture of Na_2_SiO_3_ and EDTA-Na_2_ solution reduced the risk of the incidence of *P. italicum* (20 ± 1.32%) and the disease severity of *P. digitatum* (18.76 ± 1.32 mm), *P. italicum* (30.56 ± 9.59 mm), and *C. gloeosporioides* (9.78 ± 0.30 mm). No fruit decay was observed under treatment of the mixture. These results suggest that Na_2_SiO_3_ and EDTA-Na_2_ treatment can effectively control the four citrus post-harvest diseases.

### 3.9. Fruit Quality

As shown in Table 3, EC_50_ Na_2_SiO_3_ and EDTA-Na_2_ treatments resulted in lower weight-loss rates of fruit than the control treatment, and significantly better fruit quality in terms of soluble solid, titratable acid, and VC, suggesting that EC_50_ Na_2_SiO_3_ and EDTA-Na_2_ treatments had no negative impact on the fruit quality and could be used as preservatives to replace traditional chemicals.

## 4. Discussion

In previous studies, some GRAS salts were found to be capable of effectively controlling various post-harvest diseases of fruits and vegetables [36]. For example, 2% PS, 2% SB, and 2% PSi could control anthracnose severity [7]; sodium dehydroacetate [37] and polyhexamethylene biguanide (PHMB) [38] could effectively reduce citrus sour rot; and cinnamic acid (CA) could decrease the incidence of blue mold caused by *Penicillium italicum* in “Orah” mandarin during storage [39]. However, there have been no reports on GRAS salts with broad-spectrum inhibitory effects and their inhibition mechanism on the growth of citrus post-harvest pathogens. The Na_2_SiO_3_ and EDTA-Na_2_ reported in this study may provide more options for the prevention and control of citrus post-harvest diseases.

In fact, Na_2_SiO_3_, an inexpensive GRAS salt, has been widely used to control the postharvest diseases of muskmelon and grape [40]. However, there has been no study and application of Na_2_SiO_3_ in controlling citrus post-harvest diseases. The *in vitro* experiments in this study showed that the hyphal growth and spore germination of four pathogens could be almost completely inhibited by Na_2_SiO_3_ at a concentration of 1%, which is lower than that of PS, SB, and Psi (2%) used for controlling *C. gloeosporioides* [7]. Na_2_SiO_3_ treatment significantly reduced the disease incidence of blue mold and sour rot in citrus fruit compared with the control, as well as decreased the disease severity of *P. italicum*, *P. digitatum*, *G. citri-aurantii*, and *C. gloeosporioides*. It has been reported that Na_2_SiO_3_ increases the resistance of muskmelon and grape fruit against post-harvest diseases by activating reactive oxygen species metabolism and phenylpropanoid pathway, thereby maintaining the post-harvest quality of fruit [40]. This study demonstrated that Na_2_SiO_3_ decreased the incidence and disease severity of citrus postharvest diseases by inhibiting the hyphal growth of pathogens, providing a theoretical foundation for the application of Na_2_SiO_3_ to control citrus postharvest diseases as an important alternative.

EDTA-Na_2_ is a stable food additive used in the food industry, but there are no reports about its application in the control of post-harvest diseases in fruits and vegetables. In this study, we demonstrated for the first time the antifungal ability of EDTA-Na_2_ to control citrus post-harvest pathogens *in vitro*. Notably, the combination of EDTA-Na_2_ and Na_2_SiO_3_ decreased the sour rot incidence to 0 in vivo, as well as significantly reduced the lesion diameter of green mold, blue mold, and anthracnose, indicating that the combination has a better inhibitory effect on the pathogen than EDTA-Na_2_ and Na_2_SiO_3_ alone.

The cell wall plays an important role in maintaining the morphology and integrity of the cells [35,36,41]. In this study, the CFW and PI staining results revealed that some spores had no blue fluorescence (Figure 5A) and all hyphae had more visible red fluorescence (Figure 6A) after EDTA-Na_2_ and Na_2_SiO_3_ treatments compared with the control, suggesting that the treatments have nearly no effect on the cell wall, but disrupt the cell membrane integrity of the post-harvest pathogens. In addition, EC_50_ Na_2_SiO_3_ and EDTA-Na_2_ treatments increased the OD_600nm_, suggesting that the treatment might severely disrupt the cell membrane integrity and increase the membrane permeability, leading to nucleic acid leakage, which is consistent with the results obtained from PI staining. Moreover, Na_2_SiO_3_ treatment resulted in much higher OD_600nm_ values of *P. digitatum*, *P. italicum*, *G. citri-aurantii*, and *C. gloeosporioides* than EDTA-Na_2_ treatment, indicating that Na_2_SiO_3_ causes further damage to the cell membrane. Notably, Na_2_SiO_3_ caused the most significant damage to the cell membrane of *G. citri-aurantii* (Figure 6B).

LDs are involved in regulating the balance of lipid metabolism by changing the LD size and number [42,43]. Furthermore, in a previous study, a significant increase in LD biogenesis was observed under rapamycin treatment in *Magnaporthe grisea*, *Botrytis cinerea*, *Fusarium oxysporum*, *Fusarium annularis*, *Alternaria alternaria,* and *Fusarium graminearum* [32]. In this study, clearly visible green fluorescence was observed under Na_2_SiO_3_ and EDTA-Na_2_ treatments, indicating that Na_2_SiO_3_ and EDTA-Na_2_ induce the accumulation of LDs of *P. digitatum*, *P. italicum*, *G. citri-aurantii*, and *C. gloeosporioides*. These results indicate a potential future research direction to study the inhibition mechanism of GRAS salts against citrus post-harvest pathogens.

During citrus post-harvest storage, fruit quality traits, including cumulative weight-loss rate, soluble solid, and titratable acid, are usually determined before and after treatment to measure the applicability of antifungal agents [44]. So far, studies have confirmed that no GRAS salt would impair the fruit quality when used to control post-harvest decay in fruit [35,39]. In our previous study, KCl (K^+^) was found to be capable of controlling sour rot with decreasing weight-loss rate during a 90-day storage period [30]. Moreover, US FDA and the European Food Safety Authority (EFSA) have exempted the residue detection of GRAS salts in all agricultural commodities [5,24]. Therefore, considering our *in vitro* and in vivo results, EDTA-Na_2_ + Na_2_SiO_3_ treatment might present an effective approach to control the post-harvest diseases of citrus fruit.

## 5. Conclusions

The two GRAS salts, Na_2_SiO_3_ and EDTA-Na_2_, can alter the microstructure of spores and hyphal cell membrane and increase the cell membrane permeability, resulting in the leakage of nucleic acid and synergistic inhibition on the hyphal growth and spore germination of *P. digitatum*, *P. italicum*, *G. citri-aurantii*, and *C. gloeosporioides*. Moreover, the EC_50_ Na_2_SiO_3_ and EDTA-Na_2_ treatment conspicuously induced LD accumulation and reduced both disease incidence and disease severity without posing a negative impact on the fruit quality. These findings indicate Na_2_SiO_3_ + EDTA-Na_2_ treatment as a promising approach in controlling the post-harvest diseases of citrus fruit.

## Figures and Tables

**Table 1:** Inhibition rates of 17 GRAS salts (1%) on *P. digitatum*, *P. italicum*, *G. citri-aurantii*, and *C. gloeosporioides*.

Number	GRAS Salts (1%)	*P. digitatum* (B3, %)	*P. italicum* (P44, %)	*G.citri-aurantii* (AY-1, %)	*C. gloeosporioides* (NF28, %)
1	Sodium silicate	100	100	100	100
2	Ethylenediaminetetraacetic acid disodium salt	87.90 ± 0.19	100	100	100
3	Sodium benzoate	100	86.04 ± 0.45	100	100
4	Sodium diacetate	100	88.09 ± 1.01	0	100
5	Succinic acid	0	79.08 ± 0.52	89.47 ± 0.47	85.09 ± 0.19
6	Maleic acid	0	79.66 ± 0.90	90.36 ± 0.42	84.61 ± 1.03
7	Sodium carbonate	100	100	0	0
8	Sodium sesquicarbonate	0	0	100	89.58 ± 0.69
9	Sodium stearyl lactate	0	27.18 ± 1.66	18.63 ± 2.49	0
10	Adipic acid	0	0	86.34 ± 0.38	100
11	Aconitic acid	0	0	89.21 ± 0.66	83.46 ± 1.09
12	Fumaric acid	0	0	100	0
13	Calcium glycerophosphate	0	0	0	7.01 ± 1.78
14	Aspartame	0	20.33 ± 3.70	0	0
15	DL-Malic acid	0	0	89.69 ± 0.33	0
16	Citric acid	0	38.38 ± 1.78	0	0
17	Ferrous gluconate hydrate	0	91.26 ± 0.28	0	0

Note: The results are reported as the average value ± standard error.

**Table 2:** EC_50_ of Na_2_SiO_3_ and EDTA-Na_2_ on the hyphal growth of *G.citri-aurantii*, *P. digitatum*, *P. italicum,* and *C. gloeosporioides*.

GRAS Salts	Citrus Postharvest Disease	Virulence Equation (Y = ax + b)	EC_50_ (%)	EC_95_ (%)
Sodium silicate	*G. citri-aurantii*	y = 3.4498x + 16.216	0.06	0.17
	*P. digitatum*	y = 3.6899x + 17.2108	0.05	0.14
	*P. italicum*	y = 4.1557x + 18.0528	0.07	0.14
	*C. gloeosporioides*	y = 1.0819x + 7.2617	0.08	0.19
Ethylenediaminetetraacetic acid disodium salt	*G.citri-aurantii*	y = 2.9653x + 13.7919	0.11	0.39
	*P. digitatum*	y = 1.7099x + 10.2781	0.08	0.75
	*P. italicum*	y = 1.9901x + 9.5804	0.5	3.35
	*C. gloeosporioides*	y = 2.0019x + 11.3364	0.07	0.45

**Table 3:** Quality of fruit treated with Na_2_SiO_3_ and EDTA-Na_2_ during the storage of 30, 60, 80, and 90 d.

Time	Samples	Weight Loss Rate (%)	Soluble Solid	Titratable Acid	VC (mg/100 g)
30 d	CK	0.64 ± 0.06 ^AC^	14.00 ± 0.00 ^AC^	0.83 ± 0.00 ^AC^	0.66 ± 0.01 ^AC^
	Na_2_SiO_3_	0.56 ± 0.04 ^aB^	13.50 ± 0.00 ^aB^	0.98 ± 0.02 ^aB^	0.64 ± 0.04 ^AB^
	EDTA-Na_2_	0.52 ± 0.03 ^bc^	13.43 ± 0.06 ^Bc^	0.76 ± 0.01 ^bc^	0.65 ± 0.02 ^Bc^
60 d	CK	2.41 ± 0.20 ^AC^	13.67 ± 0.06 ^AC^	0.84 ± 0.06 ^AC^	0.62 ± 0.01 ^AC^
	Na_2_SiO_3_	2.29 ± 0.17 ^aB^	15.20 ± 0.10 ^aB^	1.06 ± 0.18 ^AB^	0.74 ± 0.05 ^aB^
	EDTA-Na_2_	2.13 ± 0.15 ^bc^	13.90 ± 0.00 ^bc^	0.79 ± 0.04 ^bC^	0.67 ± 0.01 ^bc^
80 d	CK	4.99 ± 0.42 ^AC^	13.45 ± 0.25 ^AC^	0.75 ± 0.07 ^AC^	0.60 ± 0.02 ^AC^
	Na_2_SiO_3_	4.88 ± 0.39 ^aB^	14.67 ± 0.06 ^aB^	0.85 ± 0.03 ^aB^	0.70 ± 0.01 ^aB^
	EDTA-Na_2_	4.49 ± 0.33 ^bc^	13.57 ± 0.21 ^bC^	0.67 ± 0.01 ^bC^	0.64 ± 0.01 ^bc^
90 d	CK	6.19 ± 0.53 ^AC^	14.07 ± 0.06 ^AC^	0.77 ± 0.03 ^AC^	0.58 ± 0.01 ^AC^
	Na_2_SiO_3_	6.10 ± 0.19 ^AB^	14.57 ± 0.06 ^aB^	0.89 ± 0.01 ^aB^	0.69 ± 0.01 ^aB^
	EDTA-Na_2_	5.65 ± 0.43 ^bc^	14.17 ± 0.15 ^bC^	0.75 ± 0.01 ^bc^	0.62 ± 0.01 ^bc^

Note: CK: control group; Na_2_SiO_3_: Na_2_SiO_3_ treatment group; EDTA-Na_2_: EDTA-Na_2_ treatment group. Values represent the mean ± standard deviations of three replicates. Different lower-case letters are significantly different (*p* < 0.05), different upper-case letters are significantly different (*p* < 0.01).

## Data Availability

Data is contained within the article.
